# The Effect of SiC Content on Microstructure and Microwave Heating Rate of *h*-BN/SiC Ceramics Fabricated by Spark Plasma Sintering

**DOI:** 10.3390/ma12121909

**Published:** 2019-06-13

**Authors:** Huilin Lun, Yi Zeng, Xiang Xiong, Lei Zhao, Dongling Li, Ziming Ye, Tianxiao Qian

**Affiliations:** 1 State Key Laboratory of Powder Metallurgy, Central South University, Changsha 410083, China; lunhuilin89@126.com (H.L.); xiongx@csu.edu.cn (X.X.); yeziming@csu.edu.cn (Z.Y.); qtx120313@163.com (T.Q.); 2 Beijing Key Laboratory of Metal Material Characterization, Beijing 100081, China; zhaolei@ncschina.com; 3 The NCS Testing Technology Co., Ltd., Beijing 100081, China; donglingli1973@126.com

**Keywords:** hexagonal boron nitride (*h*-BN), silicon carbide (SiC), spark plasma sintering (SPS), microwave heating

## Abstract

Hexagonal boron nitride/silicon carbide (*h*-BN/SiC) ceramics were fabricated by a spark plasma sintering (SPS) method. Phase and microstructure of ceramics were characterized and observed, respectively, using the X-ray diffraction, scanning electron microscope and electron probe microanalysis. The effect of molar ratios of SiC to *h*-BN on the microstructure, relative density, hardness, thermal conductivity, and the heating rate by microwaves on the ceramics were investigated. The results showed that the orientation of flake-like *h*-BN was significantly influenced by SiC content in *h*-BN/SiC ceramics. With the increasing of SiC content, the *h*-BN flakes gradually became an isotropic distribution from the preferred orientation aligning in a SPS pressure direction. The relative density of *h*-BN/SiC ceramics was 97.6 ± 0.9% at a molar ratio of SiC to *h*-BN of 40/60 mol%. The preferential orientation of *h*-BN flakes contributed to a relatively high thermal conductivity along the SPS pressure direction, which was beneficial to increasing the heating rate of *h*-BN/SiC ceramics in microwave fields.

## 1. Introduction

Hexagonal boron nitride/silicon carbide (*h*-BN/SiC) ceramic composite is an useful structural material possessing the advantages of *h*-BN ceramic, such as the high thermal conductivity and high chemical stability of *h*-BN [1,2,3,4,5], as well as the advantages of SiC, such as high temperature mechanical properties, ablation resistance, excellent thermal and semiconductor characteristics [6,7,8,9], which is used for thermocouple protection tubes, casting side dams, and heat sinks for semiconductor parts [10,11]. With a high heating rate and uniform sintering temperature, SiC can be used as microwave susceptor due to its high loss factor under low temperatures [12,13]. Additionally, *h*-BN has low wettability to alloy melts and low absorption rates of microwaves. Therefore, it is inferred that *h*-BN/SiC composite can be used as a heat-treatment crucible that can built for a series of temperature gradient fields, due to their inverse absorption features in microwave fields. It is believed that the crucible array of *h*-BN/SiC might be applied to the investigation of high throughput screening and heat treatment of alloys. Unfortunately, there are few reports concerning the microstructure and its effects on the heating rate by microwaves of such material.

Furthermore, the densification of *h*-BN/SiC ceramic is difficult, due to their poor sintering ability and the special flake-like structure of *h*-BN [14,15]. In order to obtain a relative high density, *h*-BN/SiC ceramic is usually sintered by hot pressing and spark plasma sintering (SPS). However, the hot pressing method is often done at a high temperature, about 1900 °C [16], and the *h*-BN grains grow rapidly, which leads to decrease of the fracture strength of ceramic. With low sintering temperatures of about 1600 °C [17], SPS is used to achieve fine grains that improve the mechanical properties. Zhai, F. et al. [17] prepared *h*-BN/SiC ceramic composites by SPS that can increase the relative density, flexural strength and fracture toughness of ceramic composites.

In this paper, *h*-BN/SiC ceramics were fabricated by SPS using the submicron powders of *h*-BN and SiC after being ball milled. The effect of molar ratios of SiC to *h*-BN on the microstructure and preferential orientation of *h*-BN/SiC ceramic are discussed. The effect of SiC content on relative density, microstructure, hardness, and especially the heating rate of ceramics in microwave fields were investigated.

## 2. Experimental

### 2.1. Materials Preparation

The powders used were *h*-BN (1–2 µm, >99.9% (wt%), Macklin Biochemical Co., Ltd, Shanghai, China), SiC (0.5–0.7 µm, >99%, Macklin Biochemical Co., Ltd) and B_2_O_3_ (74 µm, >98%, Aladdin chemistry Co. Ltd., Shanghai, China). The SiC particles were an irregular shape, while *h*-BN particles had flake-like morphology with a thickness of about 0.1 µm. The diameter-thickness ratios of *h*-BN were in the range of 10.0–20.0. The molar ratios of SiC to *h*-BN in powder mixtures were 40/60, 50/50 and 60/40 mol%. In all samples, B_2_O_3_ was added as a sintering aid and the mass ratio was 5 wt% to total mass of mixture powders. The ball-milled process used stainless-steel balls, the vessel wall, and ethanol as media for six h at 200 rpm. A planetary ball mill (MITR-YXQM-2L, Changsha MITR Instrument Equipment. Co. Ltd., Changsha, China) was used. The ball-to-powder mass ratio was 5:1. The mass ratio of ethanol to powder was 1:1. After being mixed, the slurry was dried at 60 °C for 24 h and screened through an 80 µm mesh sieve. The mixture powders were sintered by SPS equipment (Model HPS-200, Kingtier New Alloy Material Co. Ltd., Xuancheng, China) at 1600 °C for five min under a uniaxial pressure of 30 MPa. This uniaxial pressure was denoted as SPS pressure direction.

### 2.2. Microwave Heating Test

The microwave heating was tested in a microwave oven at multi-mode resonator with a microwave frequency of 2.45 GHz, and an effective heating power of 1000 W. The temperature was recorded every 10 s with temperature measurements recorded by a sheathed platinum and germanium thermocouple, which was grounded to the microwave cavity. The temperature error was ±1 °C. The total test time was 900 s and all the ceramic samples were almost the same dimensions and weight (3 mm height, 30 mm diameter, and 10 g). A refractory shield made of fibrous alumina was used for thermal insulation.

### 2.3. Characterization

The density and apparent porosity of *h*-BN/SiC ceramic composites were tested by the Archimedes method using deionized water as an immersing medium, and the relative density was calculated according to the ratio of the tested density to the theoretical density. Theoretical densities of the ceramic composites were calculated by applying the rule of mixture. The used theoretical density of SiC, *h*-BN, and B_2_O_3_ powders was 3.25, 2.27 and 2.46 g/cm^3^, respectively. The morphology was observed by a scanning electron microscope (SEM, NOVA NanoSEM230, Brno, Czech Republic) with an X-ray energy-dispersive spectrometer (EDS) analyzer. The electron probe microanalysis (EPMA) was tested by a JEOL JXA-8530F system (Tokyo, Japan). X-ray diffraction (XRD) patterns were obtained by a Model D/max 2550V instrument (Rigaku Ltd, Tokyo, Japan) at a scanning rate of 2.5°/min from 5° to 80° of 2*θ*. Micro-hardness was measured by Vickers’ indentation with a 0.98 N load for 15 s. Thermal conductivities of samples (diameter 12.5 mm and thickness 2.5 mm) at room temperature were tested by a LFA457/2/G instrument (NETZSCH, Bavaria, Germany).

To quantify the effect of SiC content on the bulk orientation, the formula was [18]:

(1) $$\mathrm{IOP}=\{\begin{array}{c} \frac{{{(I}_{100}{/I}_{002})}_{\mathrm{perp}}}{{{(I}_{100}^{\prime }{/I}_{002}^{\prime })}_{\mathrm{par}}}{,\;\mathrm{when}(I}_{100}{/I}_{002}{)}_{\mathrm{perp}}{>(I}_{100}^{\prime }{/I}_{002}^{\prime }{)}_{\mathrm{par}}\\ -\frac{{{(I}_{100}^{\prime }{/I}_{002}^{\prime })}_{\mathrm{par}}}{{{(I}_{100}{/I}_{002})}_{\mathrm{perp}}}{,\mathrm{when}(I}_{100}{/I}_{002}{)}_{\mathrm{perp}}{<(I}_{100}^{\prime }{/I}_{002}^{\prime }{)}_{\mathrm{par}} \end{array}$$

where, *IOP* is the index of orientation preference. *I*_hkl_ and *I′*_hkl_ are the intensities of diffraction peaks measured in the ceramics from the surface perpendicular and parallel to the pressure axis (SPS pressure direction), respectively. When *IOP* is ±1, the grains orientate randomly in ceramics. An *IOP* of >1 means that the *c*-axis of *h*-BN lattice tends to orientate perpendicular to the SPS pressure direction. An *IOP* of <−1 indicates that the *c*-axis prefers to be orientated to the SPS pressure direction.

## 3. Results and Discussion

**Figure 1:**
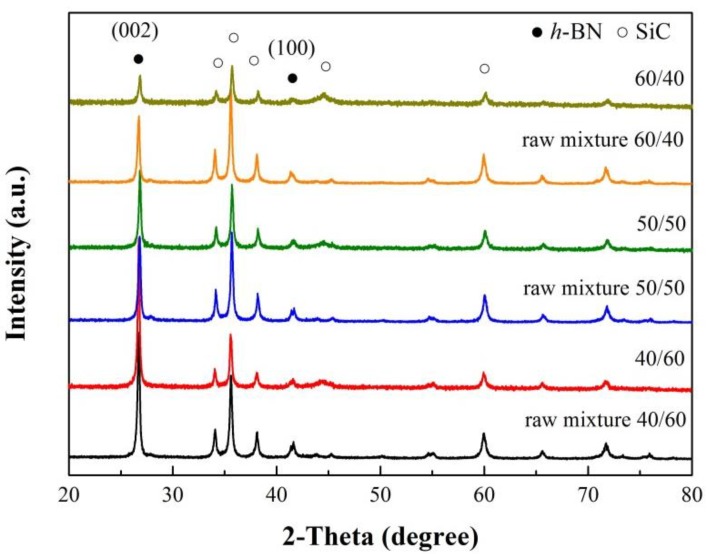
XRD patterns of mixture powders at different molar ratio of SiC to *h*-BN after ball milling.

**Figure 2:**
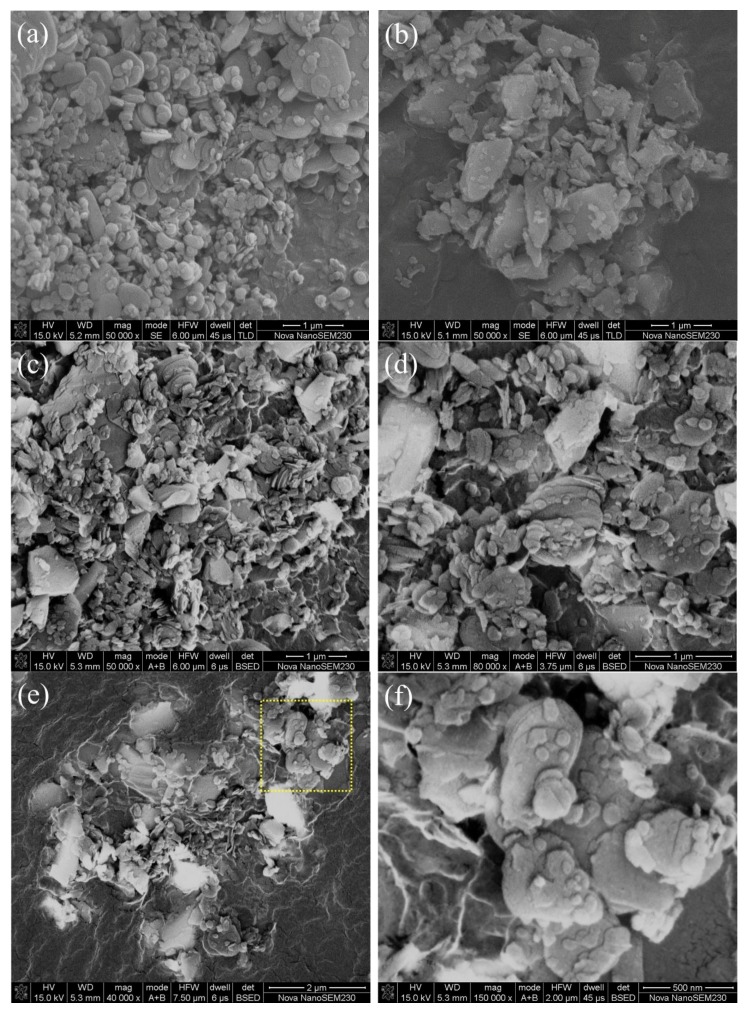
Morphologies of *h*-BN (**a**), SiC (**b**) from SEM information and BE images of ball milled powders with a molar ratio of SiC to *h*-BN of 40/60 mol% (**c**), 50/50 mol% (**d**) and, 60/40 mol% (**e**,**f**). Frame **f** is the magnified part of dotted part of frame e.

The XRD patterns (Figure 1) of mixture powder samples after being ball milled showed that all of the observed diffraction peaks were indexed to the characteristics of *h*-BN (JCPDS 73-2095) and SiC (JCPDS 49-1428). The locations of *h*-BN peaks changed slightly after ball milling which led to a decrease in the intensity and the broadening peaks of *h*-BN. Changes in (002) planes at 26.7° in particular, were attributed to the decrease in grain size and the introduction of lattice strain [19]. The contamination by Fe from the ball-mill ball and vessel might occur during the milling treatment. In this experiment, no impurity had been observed in the XRD data. In SEM backscattered electron (BE) images (Figure 2b–f), the bright particles were SiC, while *h*-BN flakes were relatively dark. The SiC particle sizes after ball milling at a molar ratio of SiC to *h*-BN of 40/60, 50/50 and 60/40 mol% were much the same as raw SiC (Figure 2b–e), no larger than 0.7 μm. The aspect ratio of the *h*-BN flake at molar ratio of SiC to *h*-BN of 40/60, 50/50 and 60/40 mol% had no obvious difference after ball milling, which was almost the same as raw *h*-BN (Figure 2a–f). It could be concluded that both the particle size of SiC and aspect ratio of the *h*-BN flakes changed little after ball milling and the effect on *IOP* value was small.

**Figure 3:**
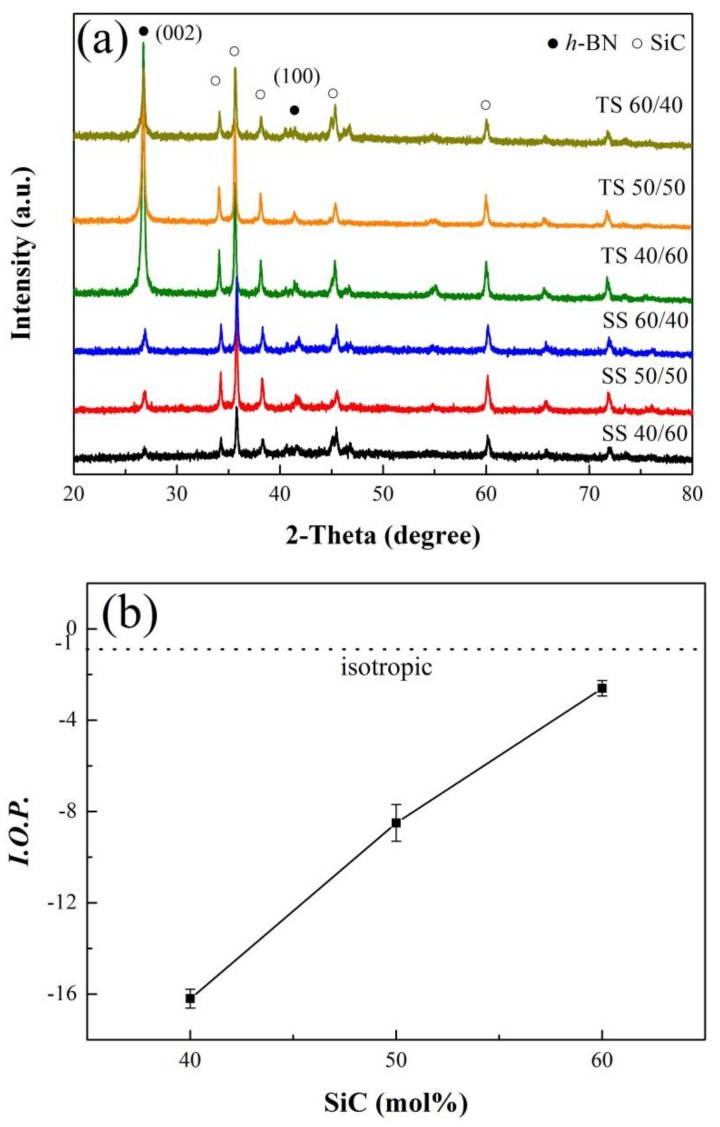
XRD patterns of the top and side surfaces of the samples prepared under different molar ratio of SiC to *h*-BN (**a**) and, the relationship between index of orientation preference (*IOP*) and different contents of SiC and *h*-BN (**b**). Error bars represent 95% confidence interval.

Figure 3a shows the XRD patterns of *h*-BN/SiC ceramic samples from top and side surfaces after SPS. The top perpendicular to the SPS pressure direction was denoted as TS, while the side surfaces parallel to the SPS pressure direction was denoted as SS. All ceramic samples showed the characteristic peaks of *h*-BN and SiC (Figure 3a). The diffraction peaks of the (002) planes, perpendicular to *c*-axis, had stronger intensity on the TS surface than on SS surface. The diffraction peak of (100) plane, parallel to *c*-axis, had a relative weak intensity on the TS surface. This suggested that the *c*-axis of the *h*-BN lattice preferred to orientate to the SPS pressure direction. With the increase of SiC content, the intensity of the (002) peak on the TS surface decreased dramatically, while the intensity of the (002) peak on the SS surface increased. This indicated that high a content of SiC, caused the *c*-axis of the *h*-BN lattice to grow perpendicular to the SPS pressure direction.

The relationship between *IOP* and different SiC contents was shown in Figure 3b. For *h*-BN/SiC ceramic with a SiC content of 40 mol%, the *IOP* value was −16.4 ± 0.4, which suggested that the *h*-BN grains were orientated parallel to the SPS pressure direction. However, with the increase of SiC content, the *IOP* value increased. When the SiC content was 60 mol%, the *IOP* value reached to −2.6 ± 0.3 (close to −1), meaning a weak preferred orientation of *h*-BN grains, and the *h*-BN/SiC ceramics were nearly isotropic.

**Figure 4:**
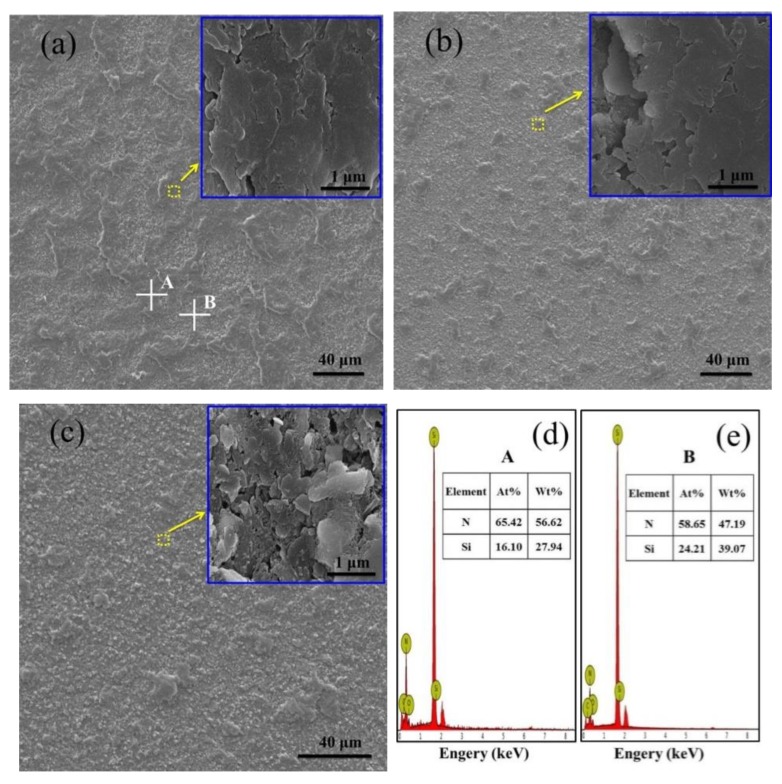
Top polished surface morphologies of *h*-BN/SiC samples. Molar ratios of SiC to *h*-BN are 40/60 mol% (**a**), 50/50 mol% (**b**) and, 60/40 mol% (**c**). **a**–**c** insets are the magnified parts, respectively. Frame **d** and **e** are the EDS spectrum of A and B point in Figure 4a, respectively.

**Figure 5:**
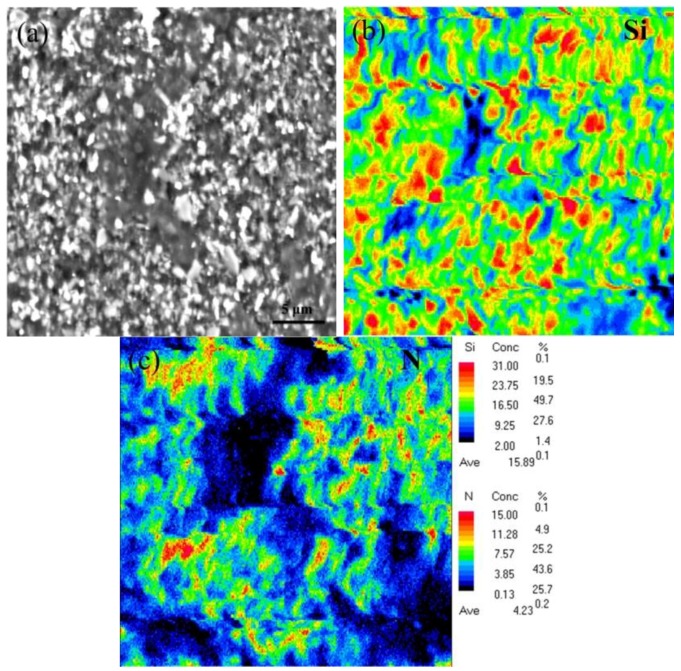
The EPMA result of Si (**b**) and N (**c**) elements distribution on the top of the polished surface (**a**).

Direct observation of the top polished surface of *h*-BN/SiC (Figure 4) showed that the typical surface could be divided into two parts with different morphology, one was a dense area (inset in Figure 4a) with dark color and the other was a less dense area (inset in Figure 4c) with porosity and light color. The morphology of the A spot was the same as magnified part inset in Figure 4a, while the morphology of the B spot was the same as magnified part inset in Figure 4c. The EDS results confirmed the existence of N and Si elements in *h*-BN/SiC samples (Figure 4d,e). There was a low Si content combined with a high content of N in the dense area (A spot), while the distribution was on the contrary in the less dense area (B spot). An increase of SiC particles resulted in the high content of Si in the less dense area, and more *h*-BN particles also led to an increase of N content. The area with an enrichment of SiC was less dense because of the poor sinterability of SiC. As the content of SiC increased from 40 to 60 mol%, the area with less density in the area of SiC enrichment became gradually larger, resulting in the low density of *h*-BN/SiC composites. The EPMA result of Si and N distribution on the top surface showed that the brighter particles were SiC particles (Figure 5). The phases of SiC and *h*-BN were distributed in the ceramic.

**Figure 6:**
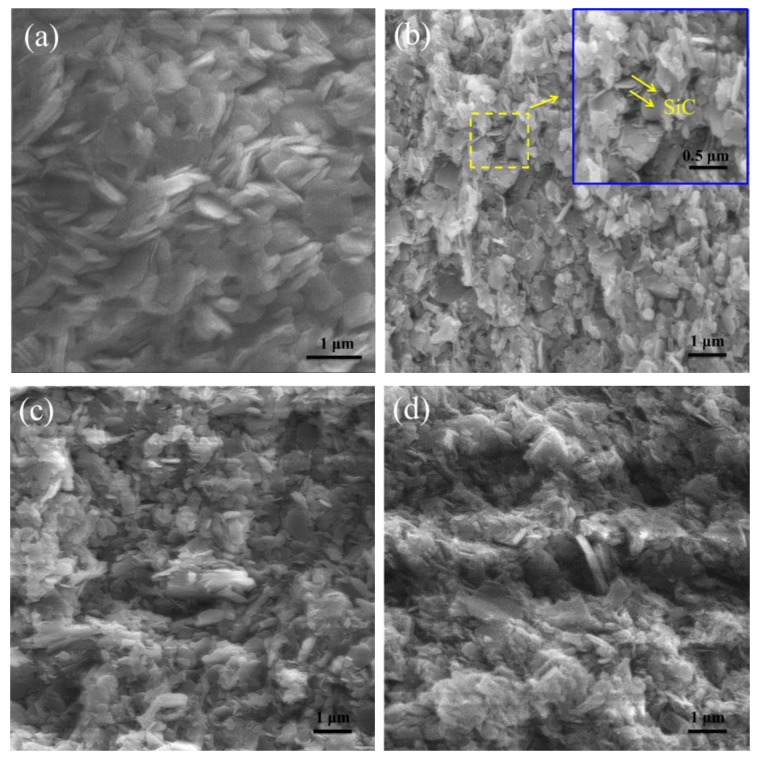
Fracture side surface morphologies of pure *h*-BN (**a**) and *h*-BN/SiC samples. Molar ratios of SiC to *h*-BN are 40/60 mol% (**b**), 50/50 mol% (**c**) and 60/40 mol% (**d**). **b** inset is the magnified part.

On the fracture side surface morphologies (Figure 6b), SiC particles were observed in 40 mol% SiC samples, but not in pure *h*-BN samples (Figure 6a). The *h*-BN grains were observed with 0.1 µm in thickness and were 1–2 µm in diameter. In the sample of 40 mol% SiC, a lot of the *h*-BN grains oriented in the same direction. Some SiC of small particles size occurred at the junction of *h*-BN grains (inset in Figure 6b). In the sample of 60 mol% SiC, the particles of SiC and *h*-BN were crossed and stacked like an iostropic structure, which was in agreement with the *IOP* calculation results. All the fracture surfaces of *h*-BN/SiC composites were uneven, indicating a typical intergranular fracture, and the roughness decreased with increased SiC content (Figure 6c,d).

**Figure 7:**
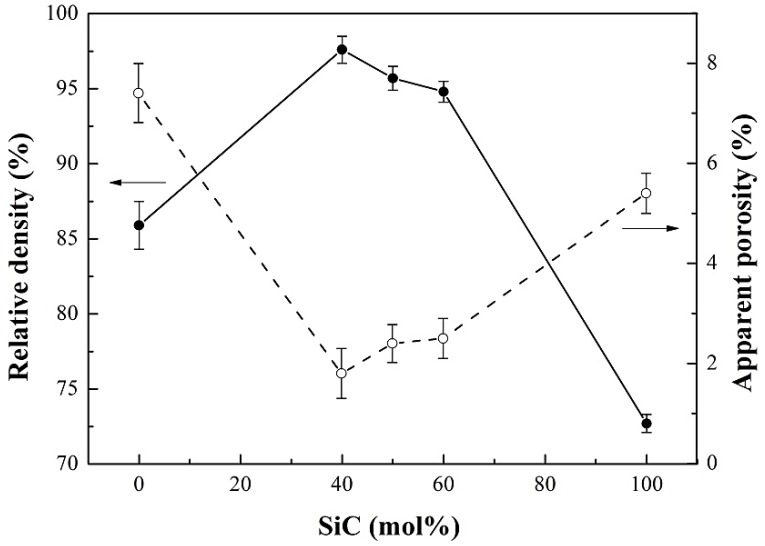
Relative density and apparent porosity of *h*-BN/SiC samples with different SiC content. Error bars represent 95% confidence interval.

**Figure 8:**
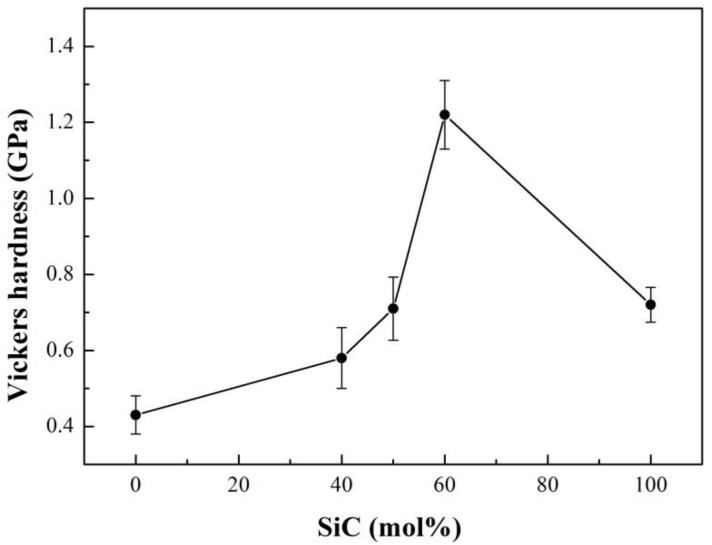
Vickers hardness of *h*-BN/SiC samples with different SiC content. Error bars represent 95% confidence interval.

The relative density increased at first and gradually decreased with an increase of SiC content (Figure 7). The relative densities of *h*-BN/SiC composites were higher than that of pure *h*-BN and SiC ceramics, and the peak was 97.6 ± 0.9% at 40 mol% SiC. Therefore, SiC particles had a significant effect on the densification of *h*-BN/SiC composites. During the densification, the “card” structure tended to be generated by flake-like *h*-BN grains and resulted in pores. With SiC particles filling in the pores, the densification of *h*-BN particles could be promoted. However, due to the poor sinterability of SiC, an excess content of SiC (more than 40 mol%) would reduce the densification of *h*-BN/SiC composites. A minimum apparent porosity of *h*-BN/SiC composites of about 1.8% was achieved at 40 mol% SiC (Figure 7). The apparent porosities of *h*-BN/SiC composites were lower than that of pure *h*-BN and SiC ceramics. The pores formed by *h*-BN could be filled by SiC particles, resulted in promoting the densification of *h*-BN/SiC composites, which had great agreement with the relative density result. The Vickers hardness of *h*-BN/SiC composites increased with the increase of SiC content (Figure 8), which was higher than that of pure *h*-BN ceramics, due to the high hardness of SiC compared to *h*-BN. Under the sintering conditions (1600 °C for 5 min under 30 MPa) in this experiment, the pure SiC sample was difficult to sinter to form dense bulk and with high porosity due to its poor sinterability, resulted in reducing its hardness. The Vickers hardness of the molar ratio of SiC to *h*-BN at 60/40 mol% reached about 1.20 ± 0.09 GPa, which was higher than that of pure SiC ceramic.

**Figure 9:**
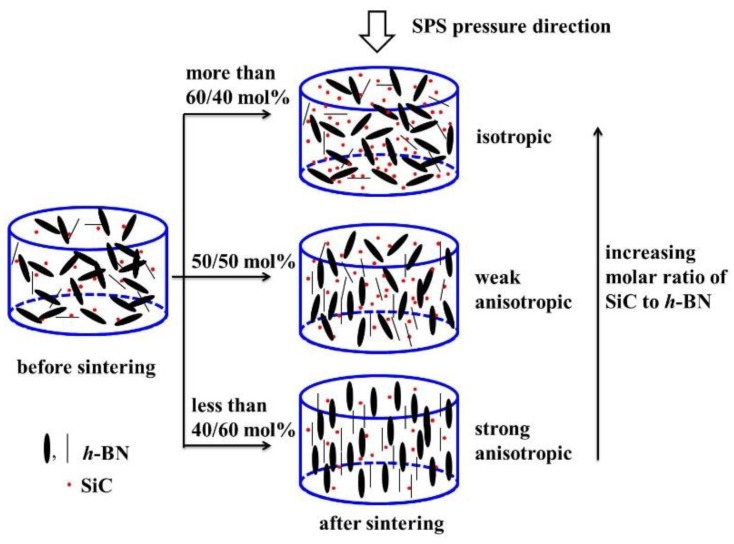
Schematic diagram of the relationship between property and different molar ratios of SiC to *h*-BN.

A schematic of the relationship between property and SiC content was summarized (Figure 9). A textured microstructure of *h*-BN/SiC composites could be fabricated at a relative low temperature (1600 °C) and moderate pressure (30 MPa). The crystal orientations of *h*-BN grains were various from the change of SiC contents. The *c*-axis of *h*-BN lattices were aligned to the SPS pressure direction because of pressure-induced preferential growth [20]. There were two main effects that resulted from the axial pressure during SPS. The first one, “the grow effect”, increased contact between *h*-BN grains, and the growth of *h*-BN grains could be accelerated along this SPS pressure direction. The second one, “the rotate effect”, was the rotation of *h*-BN perpendicular to the SPS pressure direction. The arrangement of the *h*-BN flakes was decided by a competition of these two effects. An axial pressure of 30 MPa greatly improved contact of *h*-BN grains along the SPS pressure direction and made “the grow effect” stronger than “the rotate effect”. As a result, the flake-like *h*-BN grains grew preferentially along the SPS pressure direction. For the 40 mol% SiC sample, a small amount of SiC particles distributed around of *h*-BN grains, which had no obvious effect on the contact of *h*-BN grains. The orientation of *h*-BN phases in *h*-BN/SiC composite ceramic remained parallel to the SPS pressure direction with an anisotropic texture. However, for higher SiC content samples, the intersection of *h*-BN was filled by a large amount of SiC particles, which hindered the *h*-BN grains movement and contact with each other, weakening “the grow effect” of *h*-BN. With a high SiC content (more than 60 mol%), the orientation of *h*-BN phase was weak.

**Figure 10:**
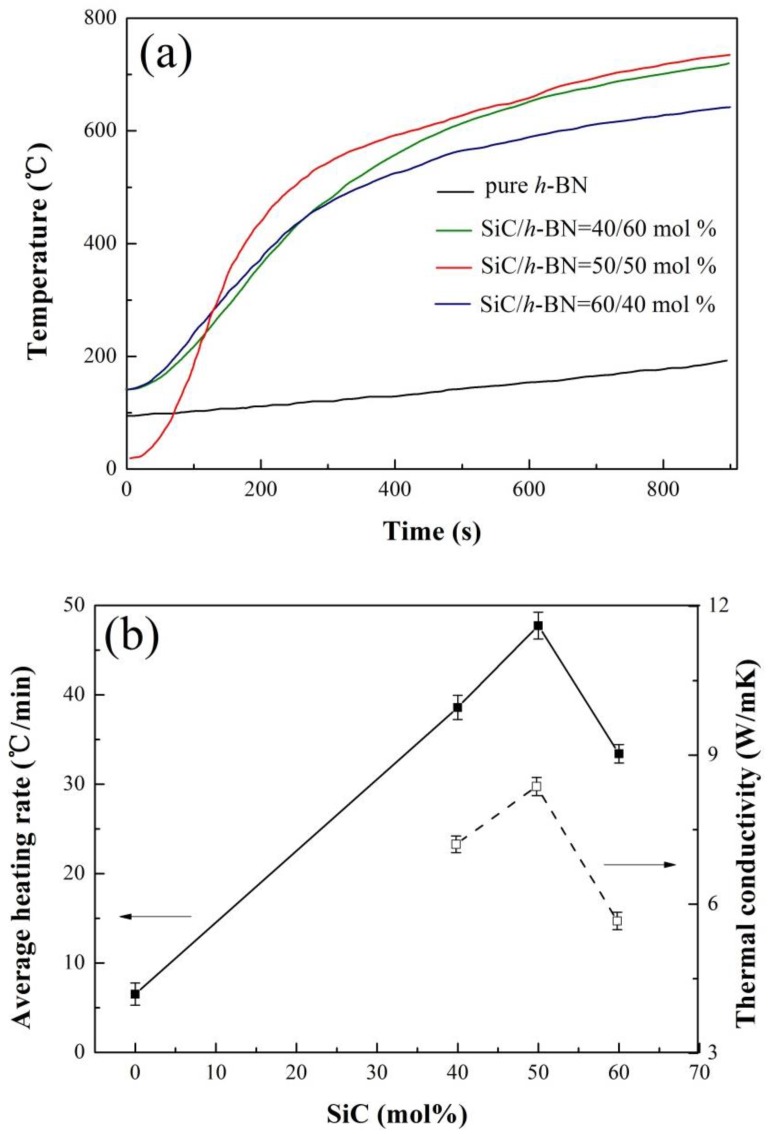
The heating curve (**a**), average heating rate and thermal conductivity (**b**) of different molar ratio *h*-BN/SiC composites in microwave fields. Error bars represent 95% confidence interval.

A high heating rate of *h*-BN/SiC composites occurred in microwave fields (Figure 10a), while the average heating rate of pure *h*-BN ceramic was low. At normal temperatures, SiC was of typical semiconductor materials with a high dielectric constant that could absorb microwaves to be heated up rapidly. The average heating rate of the 40 mol% SiC sample was about 38.6 °C/min. With the increasing temperature, the heating rate decreased gradually, especially at the temperature of over 500 °C, as the ability of SiC to absorb microwave was very poor due to its low dielectric constant at this temperature [21]. The average heating rate of *h*-BN/SiC composites reached about 47.7 °C/min at 50 mol% SiC. It was attributed to a high ratio of SiC content contained, which showed an enhanced microwave absorption capacity, resulting in a high average heating rate. When the molar ratio of SiC to *h*-BN was 60/40 mol%, the average heating rate decreased. As the in-plane and cross-plane properties of *h*-BN were different, the value of in-plane thermal conductivity was more than 20 times larger than the cross-plane [22]. The *h*-BN/SiC composites exhibited strong anisotropy in thermal conductivities because of the orientation of *h*-BN flakes. The in-plane thermal conductivity, parallel to the SPS pressure direction, was higher than that of cross-plane (perpendicular to the SPS pressure direction). Formation of efficient thermal conductive pathways in the matrix is one of the key factors for obtaining high thermal conductivity [23,24]. The tested in-plane thermal conductivity was 7.204 ± 0.166, 8.368 ± 0.181 and 5.661 ± 0.176 W/mK at a content of SiC of 40 mol%, 50 mol% and 60 mol%, respectively. The texturing of *h*-BN flakes helped the formation of in-plane thermal conductive pathways, indicating that *h*-BN having higher thermal conductivities in-plane, would obtain more heat in unit time compared to the SiC because of its microwave absorbing property. On the contrary, the *h*-BN having lower thermal conductivities in cross-plane would get less heat. Hence, in this work, though having a high SiC content of 60 mol%, the sample still possessed a decreasing heating rate (Figure 10b) due to the low weak orientation of *h*-BN flakes (Figure 3b), and its relative low thermal conductivity. It is inferred that the preferential orientation of *h*-BN flakes is beneficial to the increasing of heating rate of SiC/BN ceramic in microwave fields.

## 4. Conclusions

The *h*-BN/SiC ceramics were fabricated by spark plasma sintering (SPS) at 1600 °C for 5 min under 30 MPa with different molar ratios of SiC to *h*-BN. The results indicated that the orientation of flake-like *h*-BN was significantly influenced by SiC content in *h*-BN/SiC ceramics. With an increasing SiC content, the *h*-BN flakes gradually become an isotropic distribution from the preferred orientation aligning in SPS pressure direction. The relative density of *h*-BN/SiC ceramics was 97.6 ± 0.9% at the molar ratio of SiC to *h*-BN of 40/60 mol%. The average heating rate was about 47.7 °C/min at 50 mol% SiC. The hardness increased with an increasing SiC content. With a relatively high thermal conductivity, the preferential orientation of *h*-BN flakes is beneficial to the increasing of heating rate of *h*-BN/SiC ceramics in microwave fields. This offered an opportunity to optimize the performance and promote wider applications for *h*-BN/SiC materials.
